# Serum iron, zinc and copper among Indian patients with leukoplakia, oral submucous fibrosis and oral squamous cell carcinoma

**DOI:** 10.6026/973206300200660

**Published:** 2024-06-30

**Authors:** Pratibha Kavle, Rashi Dubey, Annaluru Sri Sasank Tejaswee, Gunmeek Kaur, Animesh Mondal, Vishal D Patel, Keyur Jitendrakumar Patel, Ansee Rameshbhai Vaghani

**Affiliations:** 1 Department of Oral Pathology, Bharati Vidyapeeth (Deemed to be University) dental college and hospital, Navi Mumbai, India; 2 Department of Pedodontics and Preventive Dentistry, SharadPawar Dental College and Hospital(DattaMegheInstitute of Higher Education and Research (Deemed to be a University), Sawangi, Wardha, Maharashtra, India; 3 Department of Oral and Maxillofacial Surgery, Kalinga Institute of Dental Science, KIIT Deemed to Be University, Bhubaneswar, Odisha, India; 4 Department of Oral and Maxillofacial Surgery, LuxmiBai Dental College and Hospital, Patiala; 5 Deptartment of Oral and Maxillofacial Pathology, Dr.R.Ahmed Dental College & Hospital, Kolkata; 6 Department of Orthodontics and Dentofacial Orthopaedic, Dhramsinh Desai University, Nadiad, Gujarat, India; 7 Government Medical College Surat, Gujarat, India; 8 Department of Oral Pathology and Microbiology, Manubhai Patel Dental College, Vadodara, India

**Keywords:** OSMF, Leukoplakia, OSCC, serum, iron, zinc, copper

## Abstract

The serum levels of iron, zinc and copper in patients with leukoplakia, oral submucous fibrosis (OSMF) and oral squamous cell carcinoma (OSCC) and compare them with normal subjects is of interest to dentists. The effort was to determine a parameter that will aid the initial diagnosis, a more efficient therapy plan, and ultimately a better prognosis. Participants in the study comprised 40 healthy normal volunteers, 60 patients diagnosed with leukoplakia, 60 patients diagnosed with OSCC, and 60 patients diagnosed with OSMF. After fasting for the whole night, blood samples were taken from each participant. There was analysis by inductively coupled plasma-optical emission spectrometry (ICP-OES) for the determination of trace elements; iron, copper, and zinc. The serum levels of iron and zinc in normal subjects was greater as compared to patients with leukoplakia, OSMF and OSCC. There was increase in serum copper levels in patients with oral leukoplakia, OSMF and OSCC as compared with normal subjects.

## Background:

Globally, oral cancer ranks as the 6th most prevalent type of cancer. It is noteworthy that more than 90% of instances of oral cancer are oral squamous cell carcinomas (OSCCs), accounting for one-third of all occurrences worldwide [1, 2-3]. The development of oral submucous fibrosis (OSMF) and oral leukoplakia leads to OSCCs. More cases of OSMF have been reported in South and Southeast Asia, especially India, in the past few years. In contrast, North America and Europe have recorded fewer cases [4, 5, 6-7]. Because of the consumption of cigarettes and other tobacco goods, incidences of OSMF are most frequently reported in the younger demographics. Any substance containing tobacco that is ingested in a way other than smoking is referred to as smokeless tobacco [8, 9, 10-11]. Oral submucous fibrosis (OSMF) is a potentially cancerous condition that primarily affects individuals of Asian heritage [12, 13]. It is a progressive, chronic illness whose clinical manifestation is contingent upon the disease's stage at the time of diagnosis. A large percentage of patients have lip with reduced flexibility, tongue, or palate that is inflexible, which limits their ability to open their mouths and move their tongues to varied degrees [14, 15- 16]. They also typically have an antipathy to spicy foods. Submucosal fibrosis, which affects the majority of the mouth, upper third of the esophagus and pharynx is the disease's characteristics [17, 18-19].Chewing areca nuts, dietary inadequacies, inflammatory processes, and genetic susceptibility are among the etiological variables that have been proposed to initiate the illness process [20-21]. The development of OSMF has been linked to nutritional deficits, particularly in iron and vitamin content [22, 23- 24]. The overall viability and overall wellness of the digestive tracts epithelia, as well as the proper functioning of enzymes, depend on iron. Another theory links OSMF to an Asian form of sideropenic dysphagia, in which a long-term iron shortage makes mucosal allergens like areca nut derivatives more active towards irritation [21- 25]. Serum iron levels particularly are thought to be one of the biochemical markers for hemoglobin levels in nutritional evaluation [12-16]. The structural integrity of membrane in the mouth can be impacted by deficiencies in iron [13- 15]. A trace element is one that is needed in quantities that are below 0.01% of the organisms mass. But when a trace metal concentration rises too high, it becomes poisonous for all of them [6- 12]. Oral cancer is thought to be the most frequent neoplasm in emerging nations. The most prevalent potentially malignant lesion in the mouth is leukoplakia, which has a higher chance to convert malignantly over the course of subsequent years [13-15]. Because trace elements like iron, copper, zinc etc. are reported to be extensively altered in head and neck, lung, and breast carcinomas, they have garnered a lot of attention recently in the diagnosis of mouth cancer and pre-cancer. Another reliable biomarker for the onset and spread of carcinogenesis is the copper to zinc ratio [14-17]. Because many enzymes depend on trace elements for their proper function, changes in these biochemical indicators' blood levels have been related to the etiology of a number of malignancies, especially oral cancer [11-16]. As a result, the function of trace elements has been thoroughly investigated in recent years in relation to a number of cancer types, including the cirrhosis of the liver and breast cancer, gastric cancer, lung cancer, oral cancer, and pancreatic cancer [12- 19]. Thus, this essential significance was taken into consideration when the current investigation was conducted. Therefore, it is of interest to evaluate serum level of iron, zinc and copper in patients with leukoplakia, OSMF and OSCC and compare them with normal subjects. The effort was to determine a parameter that will aid the initial diagnosis, a more efficient therapy plan, and ultimately a better prognosis.

## Methods and Materials:

## Subjects and Methods:

Participants in the study comprised 40 healthy normal volunteers, 60 patients diagnosed with leukoplakia, 60 patients diagnosed with OSCC, and 60 patients diagnosed with OSMF.

## Inclusion criteria:

[1] Ages ranged from 25 to 60, regardless of gender.

[2] Category I consisted of healthy individuals who did not smoke or drink alcohol and did not have any mouth lesions;

[3] Patients having a confirmed leukoplakia diagnosis were included in category II;

[4] Patients in category III had an OSMF diagnosis that was verified.

[5] Individuals in category IV who had an official diagnosis of OSCC were part of the research.

## Exclusion criteria:

Excluded were cases with a documented history of systemic illness. In addition, individuals with mental illnesses and immuno-compromised patients were not allowed to participate in the trial, nor were patients with oral epithelial cell cancer or leukoplakia that had been treated. Additionally, subjects who took supplements containing trace elements were not included.

## Specimen collection:

The participants signed a consent form after receiving a thorough explanation of the process.

## Approach:

After fasting for the whole night, blood samples were taken from each participant. Utilizing sterile disposable syringes and all the appropriate measures to prevent hemolysis, five milliliters of blood were extracted from antecubital veins in the chosen patients. Without the use of any chemicals or anticoagulants, five milliliters of blood sample were placed into a plain red tip vein puncture tube. The specimen was subsequently allowed to coagulate at room temperature for approximately two hours before being centrifuged for ten minutes at 3000 rpm to separate the serum. The serum was immediately stored for the trace element analysis at -20°C. Analysis by inductively coupled plasma-optical emission spectrometry (ICP-OES) for the determination of trace elements; iron, copper, and zinc.

## Method of experimentation:

From the previously stored samples, 0.5 ml of sera is removed, allowed to come to room temperature, and subsequently diluted using 3 ml of milli-Q water. The diluted specimen is subsequently stored in the spectrophotometer's collecting tube, and the software program on the connected computer provides the result. After molecular dynamics (MD) analysis, the clear solution that results is measured using ICP-OES.

## Statistical analysis:

The data was presented in the form of Mean±SD. The data was subjected to statistical analysis using SPSS software version 21. Chi square test was used for statistical analysis. P value ≥ 0.05 was considered as statistically significant.

## Results:

The serum level of iron was 197.56±7.9, 173.94±6.10, 172.43±4.15 and 170.79±4.36 in normal subjects, oral leukoplakia patients, OSMF patients and OSCC patients respectively. It was observed that serum iron level was lesser in oral leukoplakia patents as compared to normal subjects. Similarly the serum iron levels were found to be reduced in OSMF patients compared to normal subjects. OSCC patients were also found to have reduced level of as compared to normal subjects. The serum level of copper in normal subjects, oral leukoplakia patients, OSMF patients and OSCC patients was 147.22 ± 23.61, 188.14 ± 27.73, 189.56 ± 27.73 and 174.49 ± 36.46 respectively. There was increase in serum copper levels in patients with oral leukoplakia, OSMF and OSCC as compared with normal subjects. The serum level of zinc was 39.30±3.85 in normal subjects, 29.50±4.91 in leukoplakia, 30.50 ± 4.91 in OSMF and 26.57±3.13 in OSCC patients. The serum levels of zinc in normal subjects were greater as compared to patients with leukoplakia, OSMF and OSCC (Table 1). On comparing the serum levels between leukoplakia patients and OSMF patents and OSCC patients there was marginal decreased in serum levels of iron and zinc in OSMF patients and OSCC patients. On contrary serum copper level was marginally increased in OSMF patients and OSCC patients when compared to leukoplakia patients. On comparing OSMF and OSCC patients, serum level of iron and zinc was slightly decreased in OSCC patients and serum copper levels was slightly increased n OSCC patents as compared to OSMF patients. The difference was however not significant (Table 2).

## Discussion:

This study was conducted to evaluate serum level of iron zinc and copper in patients with leukoplakia, OSMF and OSCC and compare them with normal subjects. The effort was to determine a parameter that will aid the initial diagnosis, a more efficient therapy plan, and ultimately a better prognosis. The findings of our study were in compliance with the other studies [11- 19]. It's possible that patients with OSMF have lower iron levels because iron is used in the production of collagen [12-21]. According to some theories, a drop in iron content causes a decrease in epithelial vascularity, which in turn causes an increase in arecoline penetration and fibrosis [13-17]. Chewing areca nuts, inadequate diet, inflammatory processes, and genetic predisposition are a few of the etiological factors associated with the onset of the condition [17-24]. Nutritional deficiencies have been associated with the development of OSMF, namely in the areas of iron and vitamin content. Iron is necessary for the overall health and viability of the epithelia lining the digestive tract as well as for the efficient operation of enzymes [18- 25]. Another idea relates OSMF to a type of sideropenic dysphagia that occurs in Asia and is caused by a chronic iron deficiency that increases the irritability of mucosal allergens such as derivatives of areca nut. In particular, serum iron levels are considered to be one of the biochemical indicators of hemoglobin levels in nutritional assessment. Deficits in iron can affect the mouth's membrane's structural integrity [19-24].

The serum level of zinc was 39.30±3.85 in normal subjects, 29.50 ± 4.91 in leukoplakia, and 30.50 ± 4.91 in OSMF and 26.57 ± 3.13 in OSCC patients. The serum levels of zinc in normal subjects were greater as compared to patients with leukoplakia, OSMF and OSCC. The observations of our study had resemblances with other studies [14- 23]. This may be the result of the malignant cells' likely increased need for zinc, which is drawn up from the serum and results in low zinc levels. Because copper and zinc have a negative relationship, a rise in copper levels may also result in a fall in zinc levels [15-21]. An element is considered a trace when it is required in amounts less than 0.01% of the mass of the organism. However, if the concentration of a trace metal gets too high, it becomes toxic to all of them [12-17]. It is believed that the most common tumor in developing countries is oral cancer. Leukoplakia is the most common potentially malignant lesion in the mouth, and it has a greater potential to progress to malignancy in the years to come [14- 20]. Trace elements, such as iron, copper, zinc, and so forth, have recently attracted a lot of attention in the detection of oral cancer and pre-cancer since it has been observed that these elements are significantly altered in head and neck, lung, and breast carcinomas [13-18].The copper to zinc ratio is another trustworthy biomarker for the start and progression of carcinogenesis [19- 23]. Since many enzymes require trace elements to function properly, variations in the blood levels of these biochemical indicators have been linked to the genesis of several cancers, including mouth cancer [11- 17]. Because of this, the role of trace elements has been extensively studied in recent years in relation to several cancers in human body [12-19]. The serum level of copper in normal subjects, oral leukoplakia patients, OSMF patients and OSCC patients was 147.22 ± 23.61, 188.14 ± 27.73, 189.56 ± 27.73 and 174.49 ± 36.46 respectively. There was increase in serum copper levels in patients with oral leukoplakia, OSMF and OSCC as compared with normal subjects.

The results of the increased copper levels were consistent with earlier studies [15- 23]. The explanation for elevated serum copper levels in leukoplakia patients may be linked to other investigations that demonstrate areca nuts' excessive copper content and their role as a significant etiological agent in the pre-cancerous patient group that includes oral sub-mucous fibrosis as well as leukoplakia [18- 26]. Numerous trace elements, including molybdenum (Mo), selenium (Se) and copper (Cu) are known to be present in gutkha. As a result, it might be involved in changes to the serum as well as salivary trace element levels. Given that copper promotes blood vessel development, expansion, and metastasis, it has been demonstrated that serum levels of copper are higher in cancer patients [21-25]. Because the serum Cu level is correlated with the stage of Hodgkin's disease, it has been suggested that this could be a tumor marker. Zinc has the ability to stop the growth of cancer cells and cause them to undergo apoptosis [18-24]. In cancer patients, high Zn supplements have been shown to be beneficial in lowering oxidative stress and enhancing immunological responses [19-25]. It has been shown that the Cu and Zn ratio (Cu:Zn) can forecast the course of tumors in individuals suffering from osteosarcoma as well as non-small cell lung cancer. According to a study, people with metastatic osteosarcoma had a greater serum Cu:Zn ratio than individuals with original osteosarcoma [14-19]. Furthermore, another study found that the Cu:Zn ratio had predictive significance comparable to that of tumor markers such carcino-embryonic antigen. Its antioxidant properties serve as a chemotherapy preventive [15-21]. According to the study individuals who have stage IV breast cancer had much higher amounts of trace elements than patients without the disease, raising the prospect of employing trace elements for cancer early detection [13-17].

## Conclusion:

Serum iron copper and zinc analysis is a harmless, low-cost method for the detection, identification, and tracking of malignant lesions such as OSCC well as pre-malignant lesions like leukoplakia and OSMF. Consequently, these measures may serve as biomarkers that offer crucial instruments for developing an appropriate diagnosis, course of therapy, and prognosis for OSCC.

## Figures and Tables

**Table 1:** Serum levels of iron, copper and zinc in normal subjects, oral leukoplakia, OSMF and OSCC

	**Normal Subjects**	**Oral Leukoplakia**	**OSMF**	**OSCC**	**P value**
Iron	197.56±7.9	173.94±6.10	172.43±4.15	170.79±4.36	0.001
Copper	147.22±23.61	188.14±27.73	189.56±27.73	174.49±36.46	0.016
Zinc	39.30±3.85	29.50±4.91	30.50±4.91	26.57±3.13	0.027

**Table 2:** Mean difference in serum levels of iron, copper and zinc in normal subjects, oral leukoplakia,OSMF and OSCC

	**Normal Subjects vs Oral Leukoplakia**	**Normal vs OSMF**	**Normal vs OSCC**	**Oral Leukoplakia vs OSMF**	**Oral Leukoplakia vs OSCC**	**OSMF vs OSCC**
**Iron**						
Mean difference	24.72	26.11	26.95	2.288	2.321	2.031
P value	0.021	0.012	0.017	0.964	0.764	0.153
**Copper**						
Mean difference	41.72	42.49	26.16	14.44	14.53	15.37
P value	0.014	0.016	0.021	742	0.215	0.541
**Zinc**						
Mean difference	9.14	9.95	13.56	1.04	3.13	4.15
P value	0.013	0.024	0.003	0.711	0.923	0.571
